# New strategies for mitigating undesirable odors connected to geosmin and 2-MIB in minced fish muscle: a model system approach

**DOI:** 10.1038/s41598-026-51482-2

**Published:** 2026-05-09

**Authors:** Hatairad Phetsang, Manat Chaijan, Worawan Panpipat, Ingrid Undeland

**Affiliations:** 1 Department of Life Sciences-Food and Nutrition Science, Chalmers University of Technology, Gothenburg, 412 96 Sweden; 2 Food Technology and Innovation Research Center of Excellence, School of Agricultural Technology and Food Industry, Walailak University, Nakhon Si Thammarat, 80160 Thailand

**Keywords:** Alcohol dehydration, By-products, Pomace, HS/SPME, Odorant molecules, Biochemistry, Biotechnology, Chemistry, Environmental sciences

## Abstract

Freshwater fish species commonly suffer from muddy odor problems linked to geosmin and 2-methylisoborneol (2-MIB). This study examined whether the release of geosmin and 2-MIB from spiked fish muscle could be reduced by adding poly-/oligosaccharides (pectin, carrageenan, alginate, cyclodextrins (CD)) or organic acids (ascorbic acid, isoascorbic acid, citric acid, tannic acid), as well as 5 and 10% (dw/dw) of biomasses rich in some of these molecules (apple/lingonberry press-cakes (APC, LPC) and red seaweed (*Palmaria palmata*)). All pure molecules were added at 0.18, 0.35 and 0.70% (w/w) based on ADI levels set by EFSA. Results showed alginate, carrageenan, β- and γ-CD significantly decreased geosmin and 2-MIB release by up to 90%, while pectin did not. All organic acids reduced geosmin release, while isoascorbic and citric acid prevented release of geosmin and 2-MIB; effects accompanied by pH reductions. 2-MIB release was also significantly reduced by APC and LPC. Molecular docking confirmed the experimental data that carrageenan prevented release better than alginate; and that geosmin bound to polysaccharides better than 2-MIB. This study concludes organic acids, alginate, carrageenan and CDs can be used within safe limits to prevent geosmin and 2-MIB release in fish muscle. New routes to mitigate muddy off-odor in commuted freshwater fish products were revealed, some stimulating upcycling of plant side streams leaving the food chain despite nutritional and technological functions.

## Introduction

The demand for aquatic foods is expected to increase by 15% by 2030, supplying an average of 21.4 kg per capita^1^. It is prospected that freshwater fish species, such as carp and catfish, will increase their contribution to global aquaculture production, while higher-market-value fish species, such as, salmon and trout, will grow more slowly due to higher prices and the shortage of fish meal/fish oil^2^. From a nutritional perspective, the balanced n-6/n-3 polyunsaturated fatty acid (PUFA) ratio of many farmed catfish species meets the recommendation set by WHO, and the essential amino acid content of many freshwater fish species meets the recommendation by FAO^3,4^. Further to this, freshwater fish species contain important micronutrients such as iron, zinc, calcium and vitamin A^5^.

Despite the fact that freshwater fish, particularly farmed catfish species, are affordable and nutritious, the presence of geosmin and 2-methylisoborneol (2-MIB) could discourage consumers from eating them. These semi-volatile lipophilic compounds are produced by cyanobacteria, actinomycetes, fungi and bacteria, absorbed through the fish gills and accumulated in adipose tissue^6^. Even at a concentration of 0.25–0.5 and 0.1–0.2 ppb of geosmin and 2-MIB, respectively, earthy, muddy or blue-green off-flavor issues are provided in channel catfish (*Ictalurus punctatus*)^7^. Our previous study revealed that geosmin and 2-MIB content was above its threshold in all muscle types of farmed hybrid catfish (*Clarias macrocephalus* × *Clarias gariepinus*) and both molecules were released concomitantly with storage time^8^. Another study reported that sensory panelists perceived a muddy odor after adding as little as 10% channel catfish-derived surimi to crabsticks^9^.

Tentative routes to mitigate off-flavours from geosmin and 2-MIB in post-harvest fish and fish products are to remove these molecules from the muscle matrix^10^, convert them to odorless forms and/or to molecules with other odors^11,12^, or, finally, mask them by other flavors^13^. Our previous study found that by removing lipids from hybrid catfish using the pH-shift method, geosmin was decreased below its threshold, while 2-MIB remained above^10^. The possibility to separate storage and membrane lipids from proteins during pH-shift processing, which in turn allows removal of lipophilic volatile compounds, is driven by acid-or alkali-induced protein solubilization followed by centrifugation^10,14,15^. According to Forrester et al.^11^, treating catfish fillets with citric acid might convert 2-MIB into 2-methylenebornane, however, the treated fillets were not preferred by the panelists due to sour taste. Another route to take advantage of organic acids for counteracting muddy-flavored fish is to produce a fermented fish product. Yamprayoon & Noomhorm^16^ found that after fermenting *Som Fak*; a mixture of fresh tilapia, salt, garlic, and cooked rice, for 20 days, there was an 83% reduction in geosmin. However, the market for these fish products is limited.

Besides the geosmin and 2-MIB mitigation strategies mentioned above, there is a compelling idea of chelating or binding geosmin and 2-MIB with other molecules to lower their volatility. Cyclodextrins (CD) can form complexes with various non-polar flavor molecules^17^ and have an ability to form host-guest complexes, which allows them to embed undesired fishy compounds into their structure^18^. The driving force is the replacement of high-energy water molecules in the cavity of the host CD with a hydrophobic “guest”^19^. In a gelled tilapia matrix, the addition of 3% β-CD completely retained 15 out of the 18 identified volatile compounds compared to a control without CD^20^. Based on this feature, one of our hypotheses was that β-CD, as well as other CD forms could retain GSM and 2-MIB in a fish matrix.

Other polysaccharides as alginate have also been found to impart binding of volatiles and thereby reduce their release^21,22^. Such polysaccharides could also delay volatile release by changes in physical properties of a food matrix, such as raised viscosity^23–27^. Neither of these routes have however been evaluated in relation to geosmin and 2-MIB-release from fish.

Zhang et al.^28^ found that cross-processing fish co-products with antioxidant-rich juice press-cakes or seaweed decreases lipid oxidation during and after pH-shift processing in a clean-label manner. Especially, lingonberry press cake (LPC) successfully inhibited the formation of malodorous aldehydes, including hexanal, (*E*)-2-hexenal, heptanal, octanal and 2,4-heptadienal, in alkali-made protein isolate derived from herring co-products^29^. A second hypothesis was that incorporating juice press-cakes or seaweed into a fish matrix might not only inhibit the generation of offensive odor compounds from lipid oxidation by acting as an antioxidant, but additionally could prevent the release of other offensive odor compounds, such as geosmin and 2-MIB through the presence of polysaccharides and organic acids^11,16^. This concept has to the best of our knowledge not been reported on earlier.

Based on the above, the first aim of this study was to evaluate the ability of sustained-release flavor carriers (CD´s), other polysaccharides (pectin, carrageenan and alginate) and organic acids (ascorbic acid, isoascobic acid, citric acid, and tannic acid), to mitigate the release of geosmin and 2-MIB from fish muscle. Second, juice press cakes and red seaweed, naturally rich in some of the above molecules, were tested as active ingredients in fish muscle to prevent release of geosmin and 2-MIB. In both parts, a model system consisting of washed cod (*Gadus morhua*) mince (WCM) spiked with geosmin and 2-MIB was used to mimic earthy type off-flavored catfish muscle. The WCM model has been extensively utilized for gaining mechanistic insights on lipid oxidation in fish^30,31^, due to its clean nature with primarily membranes and myofibrillar proteins^32^. Here, this model has a particular value since cod -a marine fish species- does not contain endogenous geosmin and 2-MIB. Pure compound additions were harmonized with acceptable daily intake (ADI) levels set by EFSA, and plant or seaweed biomasses were added on 5 or 10% (dw/dw) level.

## Materials and methods

### Chemicals and raw materials

Alginate (Manucol® DM) and λ carrageenan (Viscarin® GP-109 F) were provided by FMC BioPolymer (Pennsylvania, USA). Pectin from apples was purchased from Fluka BioChemika (Switzerland). Ascosbic acid was purchased from Fluka BioChemika (UK). D-isoascobic acid, citric acid monohydrate and tannic acid was purchased from Sigma-Aldrich (Steinheim, Germany). α, β and γ CD under the trade names CAVAMAX® W6, W7 and W8, respectively, were provided by Wacker Chemical Corporation (Michigan, USA). The LPC (*Vaccinum vitisidaea*), which consists of peels, leftover flesh, seeds, and stems, was provided from Grangärde AB in Sweden while the apple (*Malus domestica*) press cake (APC), which consists of peels, leftover flesh, core with seeds and stems, was obtained from Kiviks Musteri AB (Kivik, Sweden). Red seaweed (*P. palmata*) was tank-cultivated at the Sven Lovén Centre for Marine Infrastructure (Tjärnö, Sweden). Geosmin, 2-MIB and (-)-2-methyl isoborneol-d3 (2-MIB-d3) were purchased from Sigma–Aldrich (Missouri, USA).

### Washed cod mince (WCM)

WCM was prepared as described by Wu et al.^33^. Cod fillets were purchased from Landala Fisk (Göteborg, Sweden). To obtain pure white muscle, connective tissue and dark muscle (including any traces of blood) were manually removed, and subsequently, the white muscle was ground using a table top meat grinder (C/E22 N, Minerva Omega group, Italy). The cod mince was washed with cold (4 °C) solutions, i.e., Milli-Q water for the initial wash, followed by two washes with 50 mM phosphate buffer at pH 6.6, each using a volume three times the weight of the cod mince. Using a buffer at pH 6.6 in the last two washes was selected as our earlier research demonstrated that this is a typical post mortem pH of catfish muscle^34^. In the last wash, mince and solution were homogenized for 1 min at 6000 rpm using a polytron (T18 digital Ultra-Turrax, IKA, Germany). The homogenate was allowed to stand for 15 min and then centrifugated at 15,000×*g* at 4 °C for 25 min by Thermo Scientific Sorvall LYNX Superspeed Centrifuge (Thermo Fisher Scientific, Waltham, USA). The pellet was collected as WCM, packed in plastic bags, and stored at − 80 °C.

### Addition of individual molecules and biomasses to WCM

The individual molecules alginate, pectin, carrageenan, ascorbic acid, isoascobic acid, citric acid, tannic acid and CD (α, β, and γ) were prepared individually at a concentration of 5% (*w*/*v*) in milli-Q water, or 0.2 M NaOH (β-CD). All the different molecules were added to comply with the lowest ADI (5 mg/kg body weight per day), which applied to β-CDs. Calculations were based on a 100 g/portion and 70 kg body weight. To expand the study domain, and better understand the retention and release in WCM, each molecule was also added at 2x above, and 2x below ADI. Added levels of individual molecules hereby ended up at 0.18, 0.35 and 0.70% (w/w) in WCM. APC, LPC, and ground *P. palmata* which had been stored at − 80 °C were cut into small cubes, treated with liquid nitrogen, and subsequently, ground to smallest possible particle size using a mortar. The moisture content of WCM and studied biomasses, including APC, LPC and *P. palmata* powder, were found to be 86.35, 79.26, 38.48 and 76.59%, respectively. Based on these numbers, amounts needed to reach 5 and 10% (dw/dw), levels previously proven successful during pH-shift processing of herring^35^, could be calculated.

### Model system preperation

Three grams of WCM was placed in a 20 mL headspace amber vial, which was kept on ice throughout the process. Fifty µL of a solution containing both geosmin and 2-MIB was then spiked to the WCM to achieve a concentration of 1 ppb. The vial was then tightly sealed with parafilm through which a stainless steel spatula was inserted to manually stir for 3 min before allowing samples to equilibrate for 1 h. The additives (0.18, 0.35, and 0.70% of individual molecules or 5 and 10% (dw/dw) of press cake/seaweed were added individually. To study the separate effect of pH, the model system was adjusted within the range of pH 4–7 using HCl/NaOH. The study of pH employed the same methodology as above with parafilm and spatula, as well as allowing samples to reach equilibrium for 1 h. Fifty µL of internal standard (2-MIB-d3) solution was finally added to achieve a concentration of 1 ppb, followed by addition of 2 mL milli-Q water, where after the vial was sealed with a magnetic screw cap with PTFE/silicone septa. The sample vials were then analysed according to below for geosmin and 2-MIB release. All samples were prepared and analyzed in duplicate.

### Geosmin and 2-MIB analysis

Geosmin and 2-MIB analysis was done following our previously developed method (Phetsang, Panpipat, Chaijan, Panya, & Undeland, 2024) comprising headspace-solid phase microextraction (HS-SPME) coupled with gas chromatography mass spectrometry (GC-MS)^36^. A CAR/PDMS (75 μm) SPME fiber was used for extraction. A GC-MS-TQ8030 triple quadrupole (Shimadzu, Kyoto, Japan) was used for analysis. Samples were separated with a ZB-1701 capillary column (Phenomenex, 30 m × 0.32 mm, 1 μm) using helium as the carrier gas. Spitless (5 min sampling time) was use as the injection mode. The oven temperature programs were as follows: holding at 3 °C for 3 min, increase at 3 °C min^−1^ to 70 °C, raise at 10 °C min^−1^ to 200 °C, increase at 20 °C min^−1^ to 260 °C and hold for 5 min. The MS spectra were recorded in the electron ionization mode (EI) at an ionization energy of 70 eV. The mass range was 10–250 m/z. The selected ion monitoring (SIM) mode was used for the scanning m/z 97, 112, 125 of geosmin and m/z 95, 108, 150 for 2-MIB. Identification of the geosmin and 2-MIB was performed based on comparisons of their mass spectra, and retention indices (RI) with those of their authentic standards under the same chromatographic conditions. Base peak areas of m/z 95 and m/z 112 from geosmin and 2-MIB, respectively, were used. Furthermore, the peak areas of geosmin and 2-MIB were normalized based on the response of their individual controls for each ADI-level addition.

### Molecular docking

Molecular docking was performed to evaluate the interactions of geosmin and 2-MIB with poly- and oligosaccharides using AutoDock 1.5.7. The receptor models of the poly- and oligosaccharides were prepared by removing crystallographic water molecules, adding polar hydrogen atoms, and assigning Gasteiger charges. The receptors were treated as rigid molecules during the docking calculations. Grid maps were generated using AutoGrid, and a grid box encompassing the entire poly- and oligosaccharide structures and potential binding sites was constructed. Docking simulations were performed using the Lamarckian Genetic Algorithm (LGA) with 10 independent runs for each ligand–receptor system. The docking results were evaluated based on the predicted binding energy, intermolecular interactions, and optimal binding conformations to elucidate the interaction mechanisms of geosmin and 2-MIB with the poly- and oligosaccharides. Three-dimensional molecular interactions and visualizations were generated using UCSF ChimeraX.

### Statistical analysis

Data analysis was done by SPSS 23.0 for Windows (SPSS Inc., Chicago, IL, USA). Duncan’s multiple-range test was used to analyse significant differences (*p* < 0.05) among samples. Correlation coefficients (*r*) were determined using Pearson’s correlation.

## Results and discussion

### Addition of polysaccharides

**Fig. 1:**
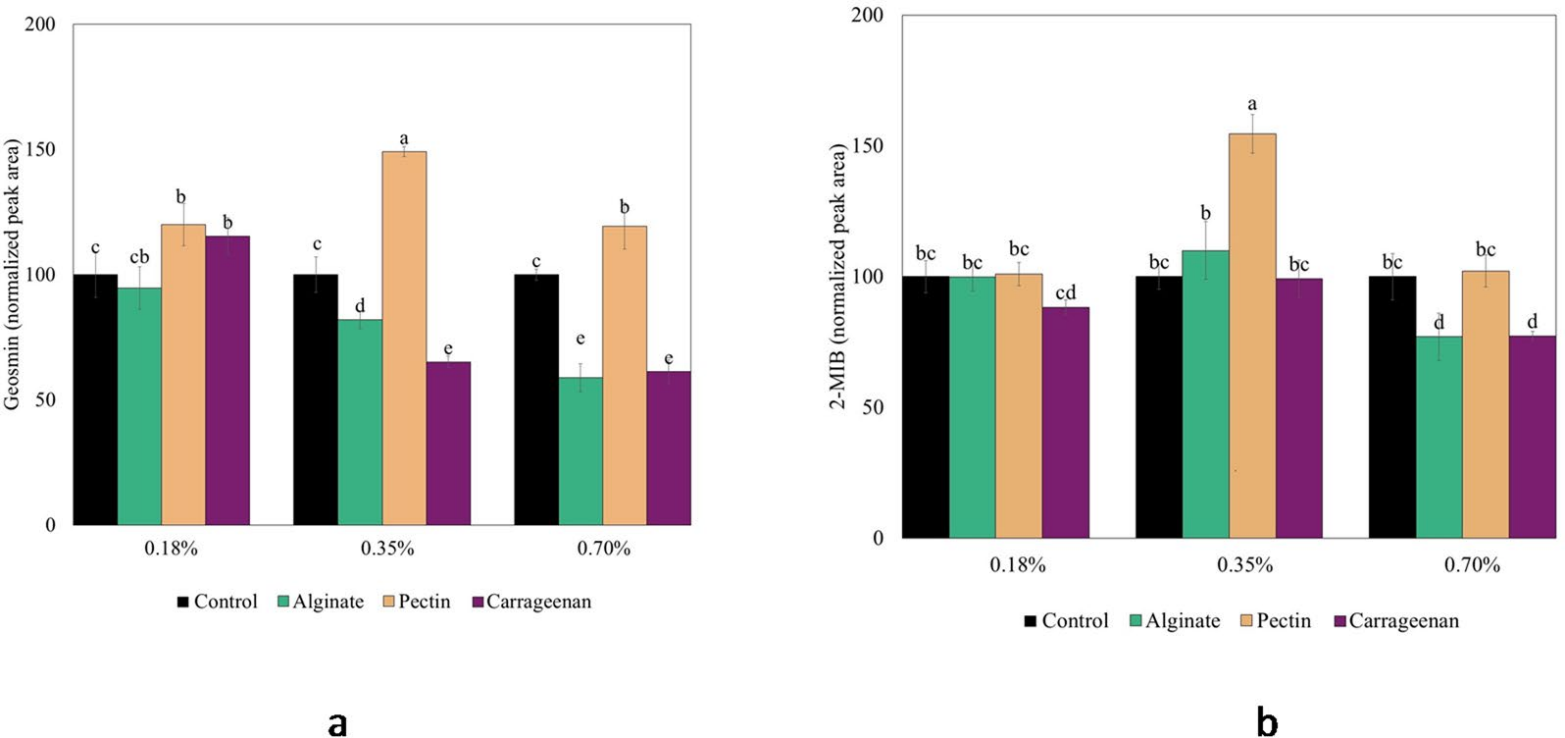
Release of (**a**) geosmin and (**b**) 2-MIB from a washed cod mince (WCM) model system (pH 6.6) as affected by adding polysaccharides (alginate, pectin and carrageenan) at the levels 2X lower than ADI (0.18%), ADI (0.35%), and 2X higher (0.7%) than ADI, which is based on an ADI of 5 mg/kg body weight per day. The peak area was normalized in relation to the peak area of the control without additions.

**Fig. 2:**
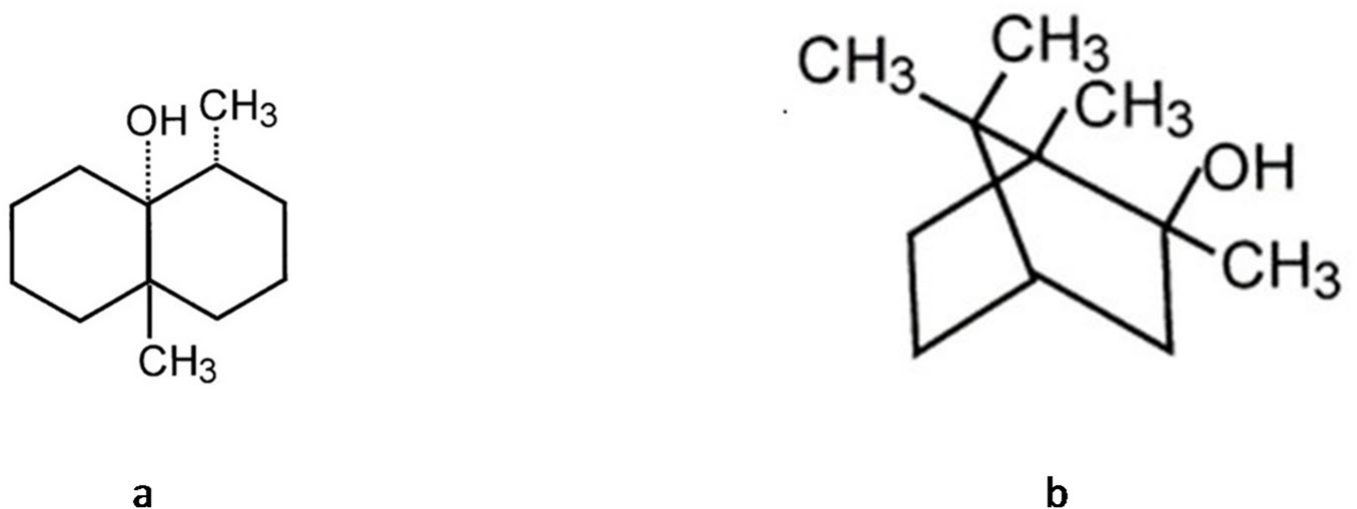
The molecular structure of geosmin (**a**) and 2-MIB (**b**).

The release of geosmin and 2-MIB from WCM after adding three different polysaccharides (alginate, pectin, carrageenan) is shown in Fig. 1a and b. Alginate and carrageenan effectively prevented geosmin release from WCM; at 0.35% the release was reduced by 17% and 35%, respectively, and when adding 0.70%, the reduction was 41% and 39%, respectively (*p* < 0.05). For 2-MIB, 0.70% of alginate and carrageenan addition could significantly prevent the release by 23% while no significant reduction was seen at 0.18 and 0.35% addition. The results thus demonstrated that carrageenan provides stronger retention of earthy–muddy odorants than alginate, with geosmin retained to a greater extent than 2-MIB (Fig. 1a and b). The lowest binding energy values from molecular docking analysis (Table 1) supported these findings, indicating stronger binding affinity of carrageenan towards both odorants compared with alginate, with geosmin showing stronger binding than 2-MIB. However, hydrogen bonding parameters (Table 1) did not correlate with the observed release behavior of geosmin and 2-MIB (Fig. 1a and b), as both carrageenan and alginate formed only one hydrogen bond with bond lengths ranging from 1.74 to 2.16 Å, suggesting relatively weak interactions. Structurally, geosmin and 2-MIB possess hydroxyl groups in similar positions (Fig. 2), indicating comparable abilities to act as both hydrogen bond donors and acceptors. Since hydrogen bonding and hydrophobic interactions are known to govern the release of odorant molecules in protein–polysaccharide systems^37^, the observed release behavior of geosmin and 2-MIB in this study is more likely governed by hydrophobic interactions. This interpretation is supported by the higher hydrophobicity of geosmin (Log *P* = 3.3) compared with 2-MIB (Log *P* = 2.7). Our findings are consistent with the work of Misharina et al.^38^, who reported that isomeric alcohols, such as aliphatic linalool and cyclic 4-terpineol, containing hydroxyl groups in similar positions, showed comparable release behavior in biopolymer matrices composed of polysaccharides (including carrageenan and sodium alginate), vegetable fibers, and gelatin.

**Table 1:** The docking results of geosmin and 2-MIB interacting with poly- and oligosaccharides.

Polysaccharides	Odorants	Lowest binding energy (kcal/mol)	Number of hydrogen bonds	Interacted residue	Interacted atom	Bond length (Å)
Alginate	Geosmin	− 2.89	1	MAV 4	O6B	1.965
	2-MIB	− 2.63	1	MAV 3	O6A	1.744
Pectin	Geosmin	− 3.52	1	AGA 3	O2	1.818
	2-MIB	− 3.24	1	ARH 4	O4	1.916
Carrageenan	Geosmin	− 3.70	1	BGA 1	O5	1.916
	2-MIB	− 3.65	1	BGA 1	OA24	2.159
Alpha-CD	Geosmin	− 6.50	1	GLC 2	O4	1.859
	2-MIB	− 6.11	1	GLC 5	O4	2.062
Beta-CD	Geosmin	− 5.92	1	GLC 6	O4	2.099
	2-MIB	− 5.65	1	GLC 3	O4	2.063
Gamma-CD	Geosmin	− 5.23	1	GLC 7	O4	2.123
	2-MIB	− 4.85	1	GLC 4	O4	1.936

However, our results differ from those reported by Boutboul et al.^39^, who observed lower retention of more hydrophobic volatiles (d-limonene, ethyl hexanoate, octanal, 1-hexanol) in carbohydrate-based matrices such as native, acetylated, and pre-gelatinized corn starch, maltodextrin, and extruded corn starch. This difference may be attributed to variations in matrix composition, as the present study involves a more complex system containing both myofibrillar proteins and polysaccharides, which may introduce additional hydrophobic interactions affecting odorant binding.

Further to differences in bond length and energy, viscosity changes may have influenced the results. There was a clear visual raise in the WCM viscosity after adding 0.7% alginate and carrageenan. In the study of Nie, et al.^27^, the 2.16–43.49% reduced release of 14 volatile aldehydes connected to fishy odor after adding 2% soluble kelp dietary fiber was ascribed a hindered volatile compound mass transfer from the matrix into the headspace governed by raised viscosity. To confirm the present observation, deeper studies would however be needed where the role of binding/interactions versus viscosity for volatile release can be clearly differentiated.

Pectin either increased or had no effect on the release of geosmin and 2-MIB (*p* < 0.05) (Fig. 1a and b). Moreover, no clear correlation was observed between the experimental results (Fig. 1a and b) and the computational docking data (Table 1) regarding the interactions of geosmin and 2-MIB with pectin. Thus, based on our docking results we can conclude that pectin at this low amount (< 0.70%) in WCM was unlikely to control the release behavior of geosmin or 2-MIB through hydrogen bonding or hydrophobic interactions. Mitropoulou et al.^40^ found that 1% pectin addition to wine enhanced the release of linalool. Similarly, Ayed et al.^37^ found that the presence of pectin in a dairy protein-based gel disrupted the solvation of hydrophobic odorant molecules, specifically the tertiary alcohol linalool. The mechanism outlined by the authors implied that pectin disrupts the water molecule-odorant interactions by giving electrostatic charges to water molecules, thereby modifying hydrophobic interactions and hydrogen bonds between the milk proteins and odorants^37^. Geosmin and 2-MIB are both classified as tertiary alcohols, why a similar mechanism may explain their raised release in pectin-fortified WCM (Figs. 1a and b).

**Fig. 3:**
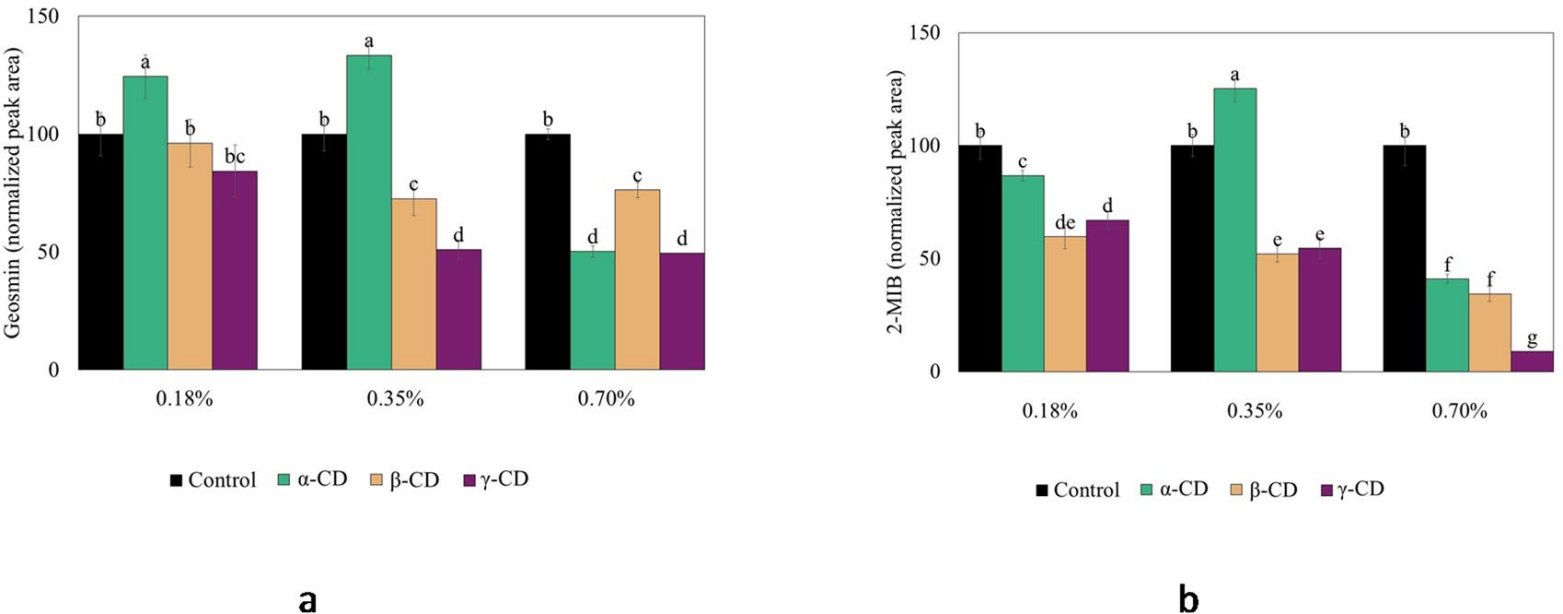
Release of (**a**) geosmin and (**b**) 2-MIB from a washed cod mince (WCM) model system (pH 6.6) as affected by adding cyclodextrins (CDs) (α, β, and γ) at the levels 2X lower than ADI (0.18%), ADI (0.35%), and 2X higher (0.7%) than ADI, which is based on an ADI of 5 mg/kg body weight per day. The peak area was normalized in relation to the peak area of the control without additions.

### Cyclodextrins

As shown in Fig. 3b, β- and γ-CD (0.18–0.7%) effectively prevented the release of 2-MIB by 33–91% (*p* < 0.05). However, for geosmin (Fig. 3a), a release-preventing effect of β- and γ-CD was observed only when adding 0.35 and 0.7%, then ranging from 24 to 51% (*p* < 0.05). For α-CD, 0.70% was needed to effectively prevent the release of geosmin and 2-MIB from WCM, by 50 and 59%, respectively (*p* < 0.05).

**Fig. 4:**
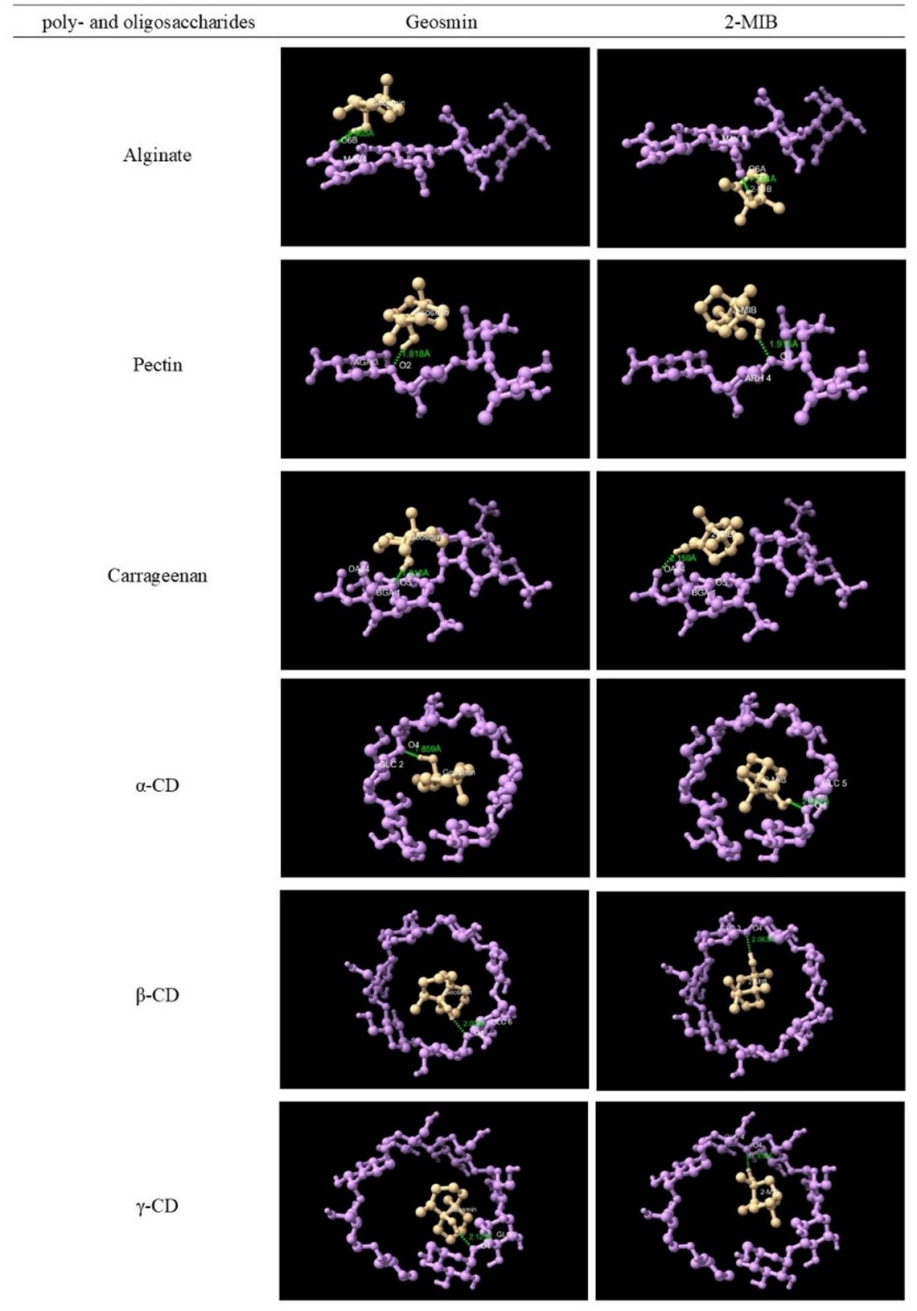
Three-dimensional diagram of geosmin and 2-MIB docking with poly- and oligosaccharides.

Inclusion capacity differences of CDs for terpenoids were previously found to be due to the steric (host-guest fitting) and hydrophobic properties of the terpenoids^41^. Evidence of potential host–guest fitting was observed in the 3D docking structures (Fig. 4). However, no consistent relationship was observed between the experimental results (Fig. 3a and b) and the computational docking data (Table 1) for geosmin and 2-MIB among the α-, β-, and γ-CD. It has been described earlier that Van der Waals forces, hydrophobic interactions and hydrogen bonds hold the CD and its guest together^42^. Both geosmin and 2-MIB are terpenes, with geosmin being a sesquiterpene (three isoprene units) and 2-MIB being a monoterpene (two isoprene units). Sesquiterpenes (β-caryophyllene and valencene) have been described to fit better than monoterpenes (γ-terpinene and carene) into CD by providing sufficient contact with the wall of the CD cavity and thus, giving a tighter encapsulation^42^. However, our results contradicted this theory since the inclusion of the sesquiterpene geosmin by CD was lower than the 2-MIB monoterpene.

In earlier comparisons among different CD´s, γ-CD was reported to have the highest inclusion capacity due to the larger size of the cavity, giving better possibility for interactions with hydrophobic terpene molecules^17^. These studies agree with experimental data shown in Fig. 3a and b, showing that γ-CD best prevented release of geosmin and 2-MIB; by 51 and 91%, respectively. β-CD is described as the most versatile CD regarding formation of inclusion complexes with terpenes by providing the strongest affinity^41,42^. This property can explain the findings of our study, showing significant release-prevention for both 2-MIB and geosmin, with the exception of geosmin at the level 2X lower than ADI. α-CD has been described to be too small to include bulky sesquiterpene (β-caryophyllene)^42^, which agrees with our findings that α-CD was the least effective CD to retain the two studied volatiles, especially the sesquiterpene geosmin.

### Organic acids

**Fig. 5:**
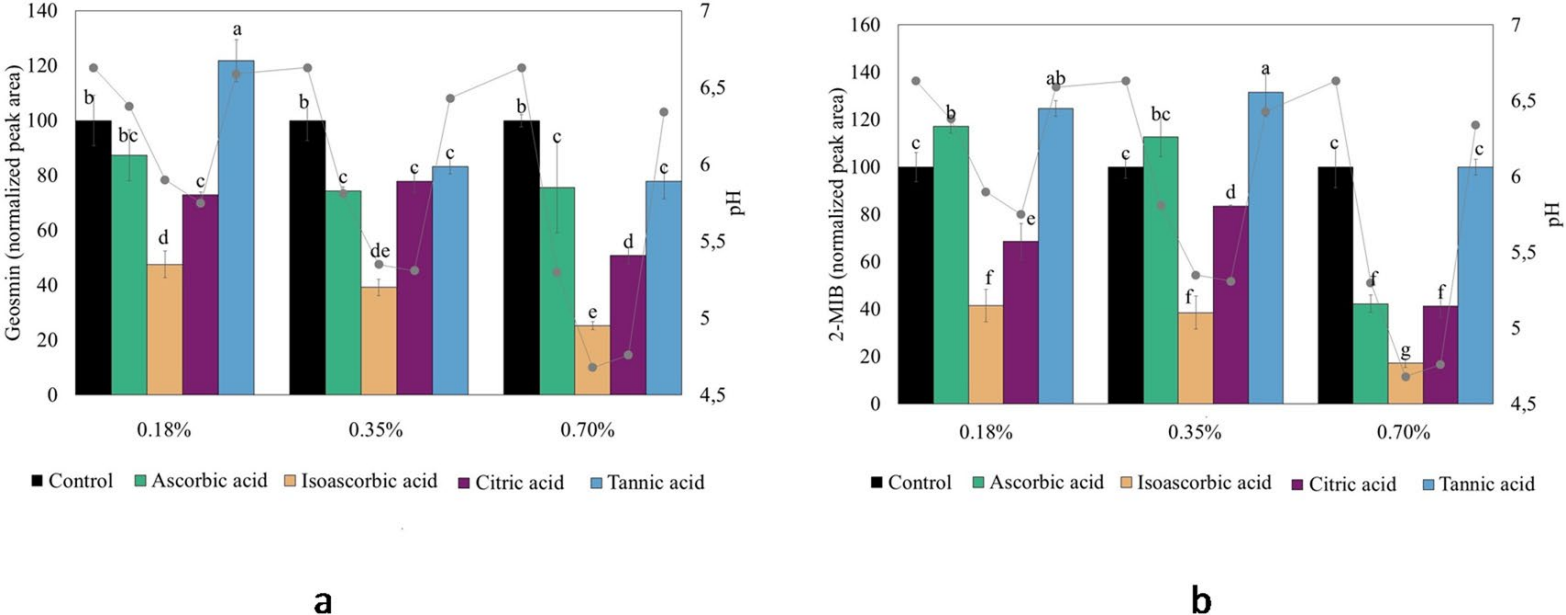
Combination chart representing the release of (**a**) geosmin and (**b**) 2-MIB as well as the pH values of a washed cod mince (WCM) model system as affected by adding organic acids (ascorbic, isoascorbic, citric, and tannic acid) at the levels 2X lower than ADI (0.18%), ADI (0.35%), and 2X higher (0.7%) than ADI, which is based on an ADI of 5 mg/kg body weight per day. The peak area was normalized in relation to the peak area of the control without additions.

The release of geosmin and 2-MIB, as well as changes in pH of the WCM model system after adding organic acids are shown in Fig. 5a and b. The addition of isoascorbic acid and citric acid (0.18–0.7%) effectively prevented the release of both geosmin and 2-MIB by 52–83% and 16–59%, respectively (*p* < 0.05). At the same time, these additions made the WCM more acidic (pH 4.68–5.90) compared to its initial pH of 6.6. Significant reductions in the geosmin release (17–75%), was also induced by ascorbic and tannic acid at 0.35 and 0.7% addition which at the same time reduced the pH of the WCM to values between 4.68 and 6.43. Ascorbic acid could also significantly reduce 2-MIB release (58%), but only after the highest addition (0.70%), which brought the pH of WCM down to 5.3. Tannic acid was found ineffective in preventing the release of 2-MIB.

**Fig. 6:**
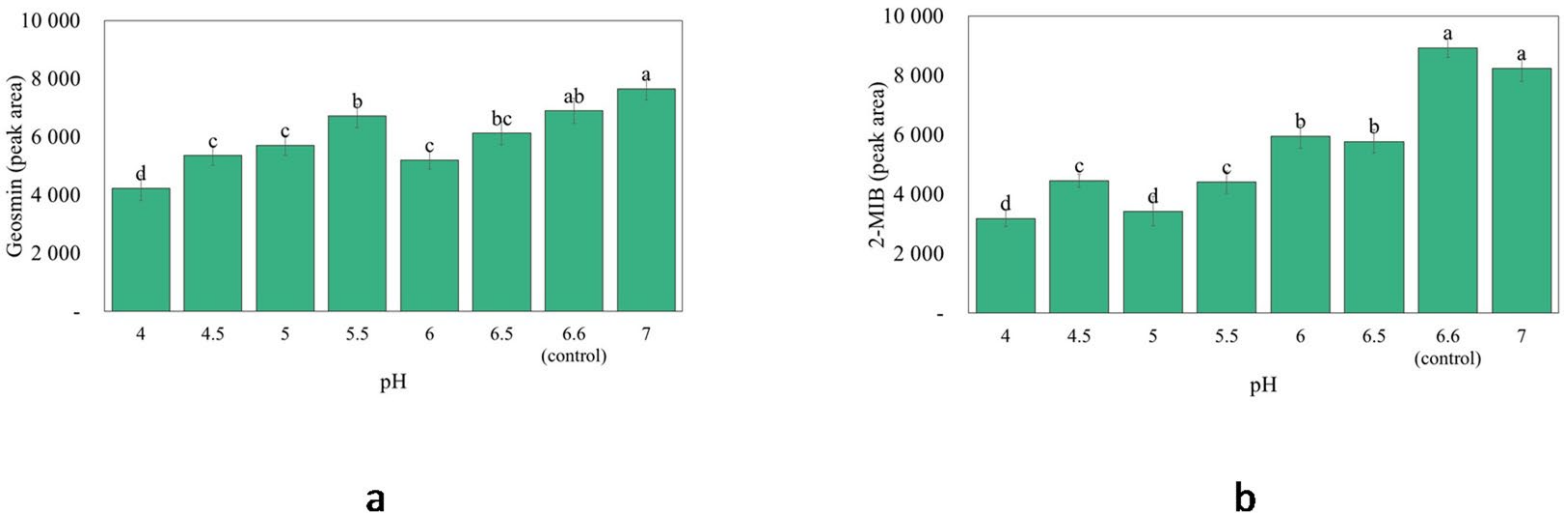
Release of (**a**) geosmin and (**b**) 2-MIB from a washed cod mince (WCM) model system adjusted to pH-values from 4–7 with HCl/NaOH.

Given the clear association between WCM pH as well as geosmin and 2-MIB release (Fig. 5a and b), a deeper investigation was done to systematically follow pH-effects over a broader range of values (pH 4–7) using HCl/NaOH (Fig. 6a and b). Here, the release of geosmin and 2-MIB had high positive correlation with the pH value of WCM, *r* = 0.82 and *r* = 0.83, respectively. That geosmin and 2-MIB peak responses decreased significantly when the pH of WCM was adjusted to values below 6.6, suggests formation of dehydrated geosmin and 2-MIB. The likely dehydration product of geosmin is argosmin, while 2-MIB can dehydrate to 2-methyl-2-bornene, 2-methylenebornane, and 1-methylcamphene^43^.

Geosmin and 2-MIB was earlier found to dehydrate in water < pH 5, but still existed at 69% and 12.6%, respectively, at pH 2.8^43^. It has also been described how dehydration of tertiary alcohols by the E1 elimination mechanism is favored under acidic conditions^44^. Such dehydration involves three steps, formation of a protonated alcohol, formation of a carbocation, and finally formation of an alkene^44^. Given that the protonated alcohol forms when accepting a proton from the acid^44^, the more protons present in the system, the more protonated alcohols can be formed. The strongest organic acid used in this study was isoascorbic acid, followed by citric, ascorbic, and tannic acid with pKa values of 2.10, 3.13, 4.04, and 8.50^45,46^. Our results revealed that the stronger the acid and the higher its concentration, the less release of geosmin and 2-MIB took place (Fig. 6a and b). Furthermore, our study found that geosmin was more prone to dehydration upon organic acid addition to WCM, as compared to 2-MIB. As previously stated, geosmin and 2-MIB share the same functional group (OH) position and should theoretically donate and accept hydrogen bonds at equal rates. Thus, more research is required to better understand the observed difference in geosmin and 2-MIB dehydration of the studied fish matrix.

### Role of juice press cakes and seaweed for volatile release

**Fig. 7:**
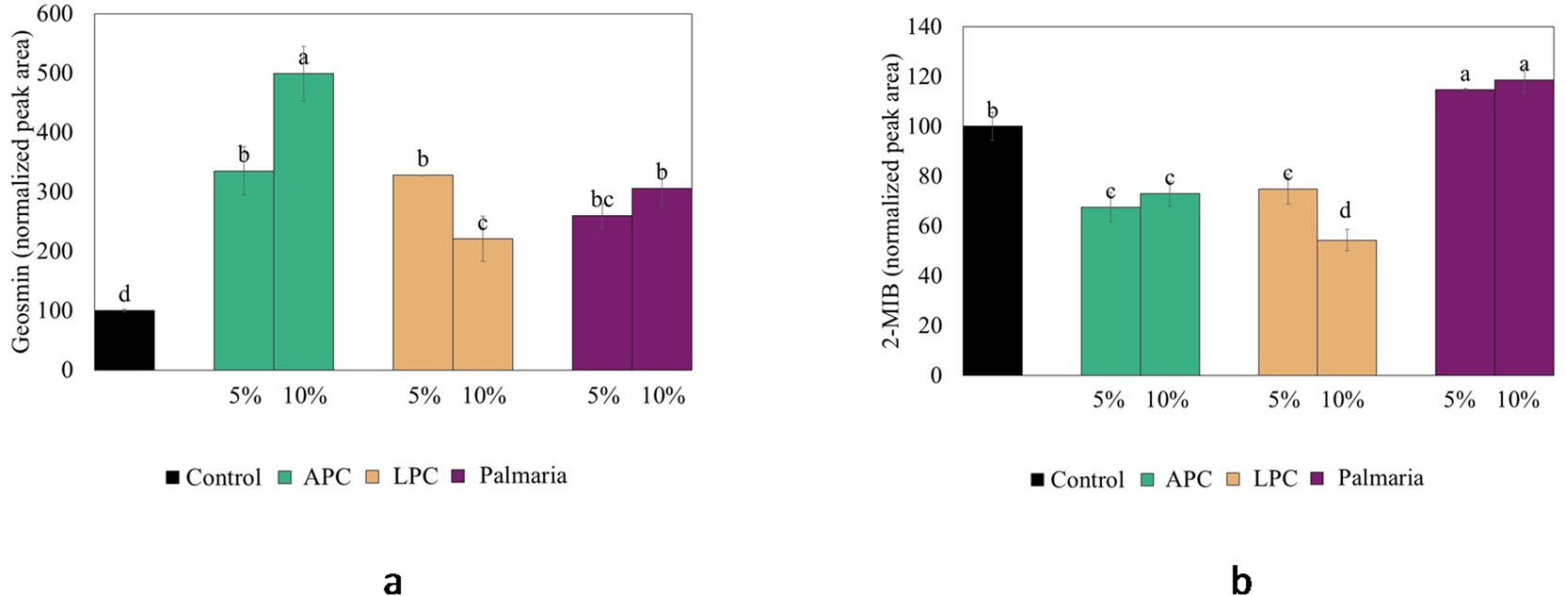
Release of (**a**) geosmin and (**b**) 2-MIB from a washed cod mince (WCM) model system (pH 6.6) as affected by adding 5% and 10% (dw/dw) of apple press-cake (APC), lingonberry press-cake (LPC), and red seaweed (*Palmaria palmata*). The peak area was normalized in relation to the peak area of the control without additions.

Lingonberry and apple juice press cakes were tested in the WCM model based on their abundance e.g. in organic acids and pectin, while *P. palmata* was selected based on high levels of polysaccharides such as xylans. However, the choice of *P. palmata* was also based on its possibility to be cultivated in Europe, as well as its red pigments and meat-like/bacon-like flavour making it an interesting food ingredient^47,48^. Figure 7a and b depict the release of geosmin and 2-MIB from the WCM model system after adding 5% and 10% of APC, LPC, and *P. palmata*. None of the studied biomasses could prevent the release of geosmin; instead the release increased significantly upon press cake or seaweed addition. *P. palmata* also increased the release of 2-MIB, possibly due to high salt levels. High ionic strength (> 1 M NaCl) has also earlier been found to limit interactions between flavor compounds and myofibrillar proteins in silver carp due to competition for charged amino acid side chain residues^49^. Opposite, adding 5 and 10% of APC or LPC significantly reduced the release of 2-MIB by 25–46%, which could be due to the lower hydrophobicity of 2-MIB (Log *P* = 2.7) compared to geosmin (Log *P* = 3.3). Earlier observations that pectin preferably causes salting out of highly hydrophobic odorants as linalool (Log *P* = 3.38) in dairy protein-based gel^37^ could partially explain the lack of effect on geosmin. Further, as both press cakes and seaweed are highly complex matrices, it is likely that there are molecules which both retain and liberate geosmin and 2-MIB from a fish muscle matrix. Beyond polysaccharides, acids and salt which are discussed already, there are also high levels of polyphenols in the studied biomasses, which could react e.g., with proteins of the WCM^50^ and with different volatiles^51^. Earlier studies of wine have shown that polyphenols can both decrease and increase volatile release^51^. This topic should be subject for further systematic studies in relation to geosmin- and 2-MIB-release.

## Conclusions

New solutions for resolving off-odor issues in commuted fresh-water fish products were tested. Results revealed that organic acids (especially isoascorbic and citric acid), alginate, carrageenan and cyclodextrins (especially β- and γ-) can be used within safe limits as additives in freshwater fish mince and surimi to prevent both geosmin and 2-MIB release. Molecular docking results suggested that the retention of geosmin and 2-MIB by carrageenan and alginate was explained by hydrophobic interactions, while CD´s acted according to an inclusion mechanism. Juice press-cakes (APC and LPC), naturally enriched in both polysaccharides and organic acids, could at 5–10% level (dw/dw) also significantly reduce the release of 2-MIB. These findings reveal that underutilized plant food side streams could be used not only for prevention of lipid oxidation^28,29,35^, but also to mitigate muddy odor release, creating a new category of hybrid foods. More research is needed to determine tentative sensorial effects of applying these molecules or biomasses to commuted products of freshwater fish species.

## Data Availability

The datasets used and/or analyzed during the current study are available from the corresponding author on reasonable request.
